# Supplementary figures and images for: Integrated Metabolomic and Transcriptomic Analyses Reveals Sugar Transport and Starch Accumulation in Two Specific Germplasms of Manihot esculenta Crantz
Source: Int J Mol Sci. 2023 Apr 13;24(8):7236. doi: 10.3390/ijms24087236 (PMC10138763; doi:10.3390/ijms24087236)

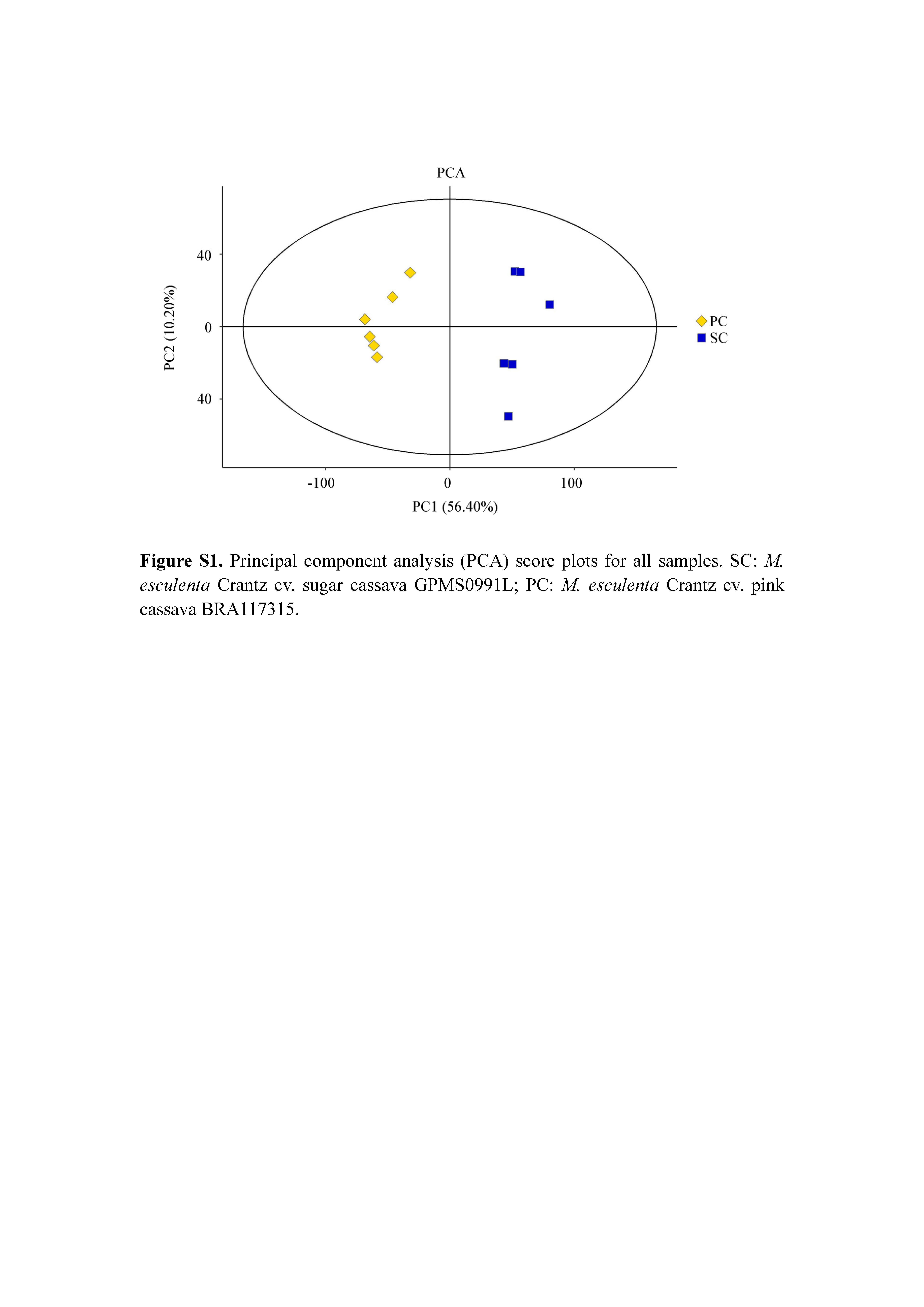

Supplement: Supplementary file 1 [file ijms-24-07236-s001.zip › Figure S1.tiff]

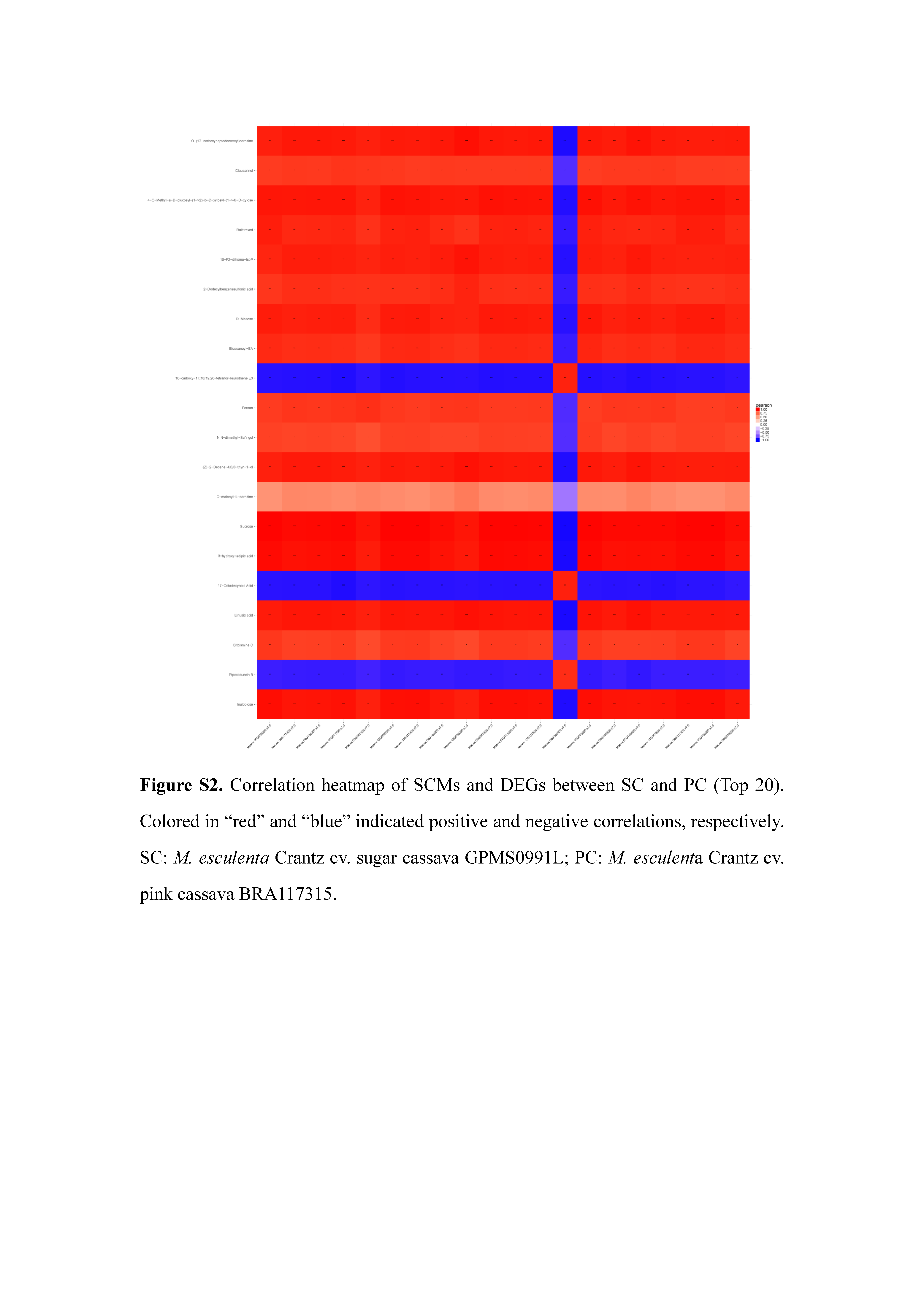

Supplement: Supplementary file 1 [file ijms-24-07236-s001.zip › Figure S2.tif]

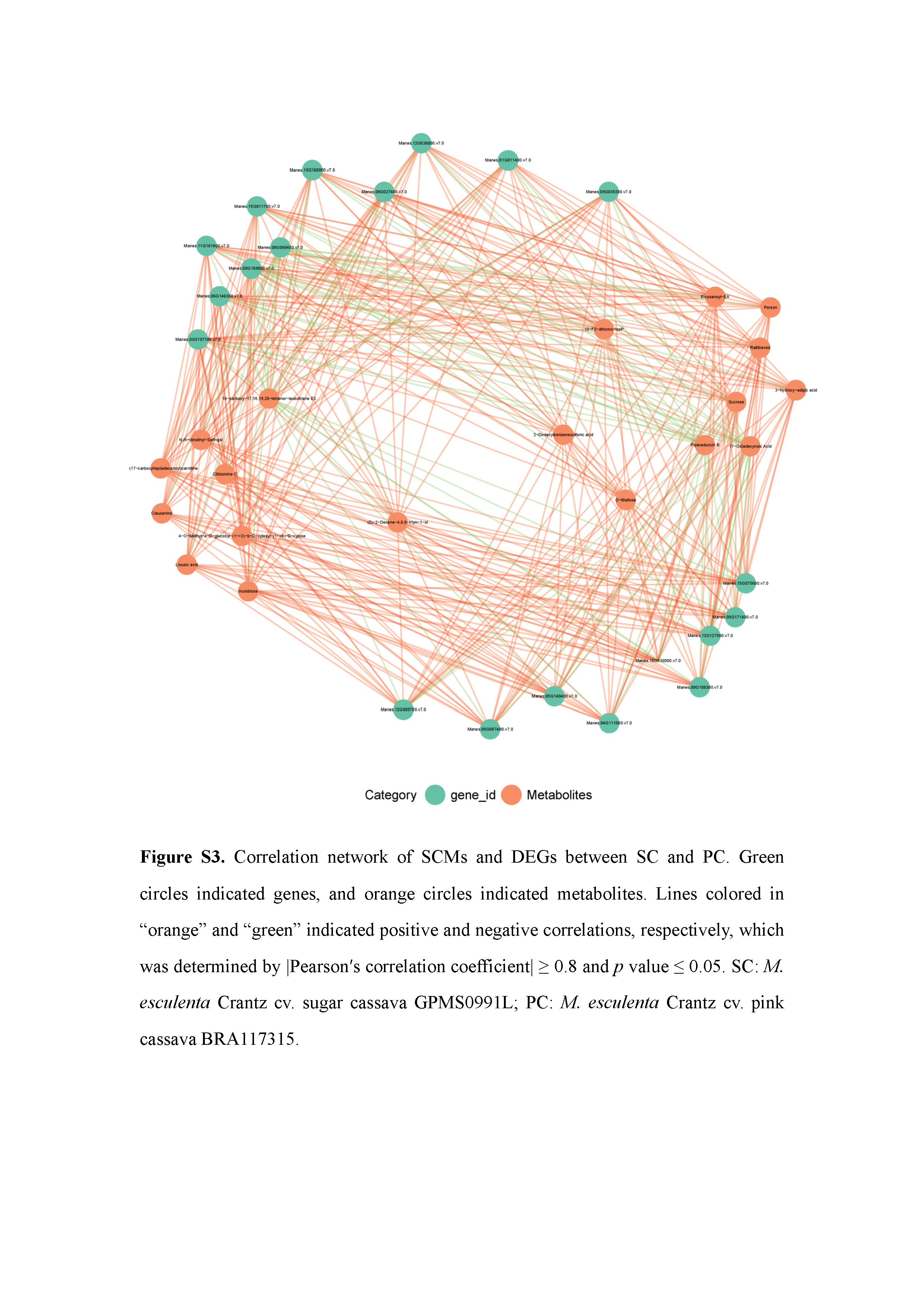

Supplement: Supplementary file 1 [file ijms-24-07236-s001.zip › Figure S3.tif]

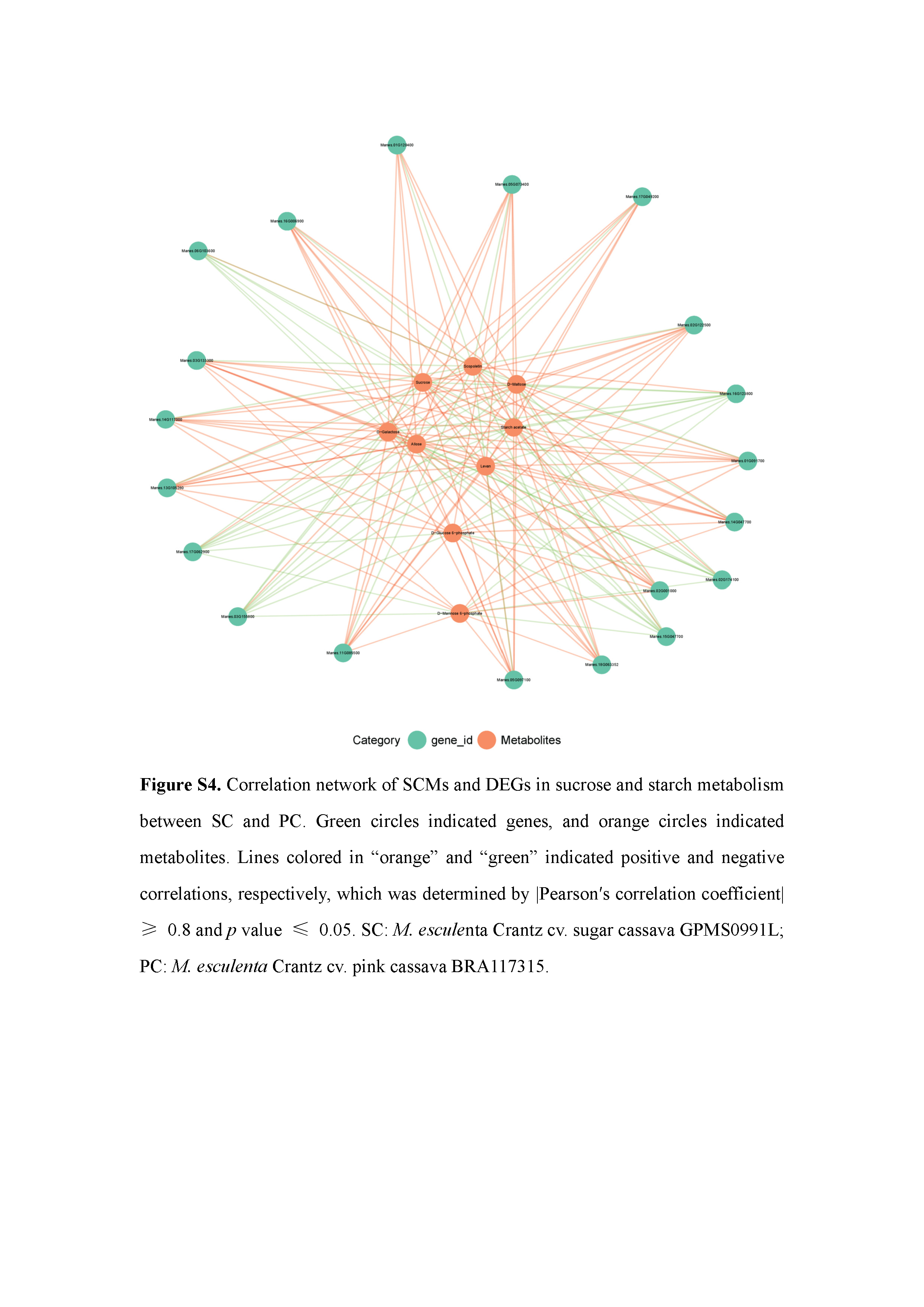

Supplement: Supplementary file 1 [file ijms-24-07236-s001.zip › Figure S4.tif]
